# Frequency of neuro-imaging in the emergency room in patients with vertigo: A cross-sectional study at a tertiary care hospital in Karachi, Pakistan

**DOI:** 10.12669/pjms.40.4.7029

**Published:** 2024

**Authors:** Mirza Noor Ali Baig, Nazir Kapadia, Ahmed Raheem, Madiha Ismail

**Affiliations:** 1 Mirza Noor Ali Baig FCPS. Aga Khan University and Hospital (AKUH), Karachi, Pakistan; 2 Nazir Kapadia FCPS. Aga Khan University and Hospital (AKUH), Karachi, Pakistan; 3 Ahmed Raheem FCPS. Aga Khan University and Hospital (AKUH), Karachi, Pakistan; 4 Madiha Ismail FCPS. Aga Khan University and Hospital (AKUH), Karachi, Pakistan

**Keywords:** Dizziness, Vertigo, Neuroimaging, CT scan, MRI, Emergency medicine

## Abstract

**Objective:**

To determine the frequency of neuro-imaging and the prevalence of positive findings in patients with vertigo visiting an emergency room of a low-middle-income-country, Pakistan.

**Methods:**

This is a retrospective cross-sectional study conducted in the emergency room of the Aga Khan University Hospital, a 550 bedded tertiary care teaching facility located in Karachi, Pakistan. The frequency of neuro-imaging in patients visiting emergency room with vertigo during 20 years (2000-2020), their findings and disposition was calculated in percentages. A cost-analysis was performed in Pakistani Rupees & US Dollars to estimate the financial burden.

**Results:**

During the emergency room visits for vertigo, neuro-imaging (CT scans, MRIs, or both) was conducted for 159 patients, accounting for 70.98% of the cases. Out of these, 64 individuals (40.25%) received a positive diagnosis, which included acute infarcts, hemorrhages, metastases, space-occupying lesions, and meningeal enhancements. Interestingly, among those with negative findings, the 98 patients faced significantly higher costs, amounting to Rs.4,108,000 ($22,449), in contrast to the positive cases, which incurred Rs.2,496,600 ($13,642).

**Conclusion:**

The frequency of obtaining neuro-imaging tests in patients with vertigo were significantly high in our study. In addition, there was a significant financial burden associated with neuro-imaging especially for our low-middle-income country.

## INTRODUCTION

Patients with vertigo or dizziness contribute a significant burden to the emergency room visits.1 The number of patients with vertigo visiting emergency rooms is on the rise, reaching in millions per year only in the United States.2 There are more than two million U.S. emergency department (ED) visits annually for dizziness or vertigo, comprising roughly 4.4% of all ED chief symptoms in awake patients.2 The data about the burden of vertigo is limited in low-income countries3 but the available literature from these countries suggest vertigo as a significant problem nonetheless. The prevalence of vertigo in India was reported to be 3.6%.4 Whereas a study conducted in Karachi, Pakistan showed the frequency of vertigo among other neurological disorders to be 3.11%.5 They are also a common reason for absenteeism from work, and disability. There are certain descriptions of the term “vertigo” in literature may refer to dizziness, light-headedness, presyncope. The symptoms can also be attributed to the change of position, for example the symptoms may be triggered by the act of movement of a head in a particular direction.6,7 Besides, the physical disability, vertigo also effects the mental health of patients, having a significant effect on their quality of life. A study reported that patients experiencing vertigo and tinnitus had a strong tendency to develop psychiatric problems including depression, anxiety and stress.8,9

The diagnostic approach to vertigo is complex, leading to a risk of misdiagnosis in patients with significant pathology.2 This is reflected even in countries with adequate resources and better healthcare facilities like the United States (U.S.), where nearly half (approximately 45,000-75,000 out of 130,000-220,000) of the patients with stroke are initially missed during their visit to the emergency rooms.10 Notably, variations in diagnostic precision exist between clinical examinations performed by emergency physicians and those performed by neurologists. Neuro-ophthalmologists developed the Head Impulse test, Nystagmus, positive Test of Skew (HINTS) examination as a bedside evaluation specifically aimed at excluding a central origin of vertigo among individuals with Acute Vestibular Syndrome (AVS). This set of physical assessments has been adopted by frontline ED clinicians and integrated into routine clinical practice. However, the examination, when used in isolation by emergency physicians, has not been shown to be sufficiently accurate to rule out a stroke in those presenting with AVS. The sensitivity and specificity of the HINTS examination performed by neurologists was 96.7% and 94.8% respectively, as compared to a sensitivity and specificity of 83% and 44% respectively when performed by a cohort of physicians including both emergency physicians and neurologists.11 This variation in the diagnostic accuracy of vertigo through clinical examination probably causes dependency of emergency physicians on specific diagnostic tests including some costly investigations like the magnetic resonance imaging (MRI). The fear of missing a significant pathology and a low probability of diagnosing etiologies like stroke on clinical examination is probably the reason for an increasing number of neuroimaging in the emergency room.

The complexity is further perpetuated when a physician must balance the risk of underutilization of relevant neurological examinations with the overuse of radiological modalities, including the computed tomography (C.T.) scans and MRIs of these patients in the emergency room. Studies show that the average cost to diagnose a patient with Benign Paroxysmal Positional Vertigo (BPPV) is 2000$ which is a consequence of multiple factors including unnecessary diagnostic tests including MRI and echocardiography, inappropriate medications, physical therapy and numerous office visits.12,13

The hospital visits of patients with vertigo impose a significant financial burden mainly because of the costs involved in neuro-imaging and admissions.2,10,14 In the U.S., this cost is estimated to exceed ten billion U.S dollar every year.10 In addition, crowded emergency rooms (E.R.s) are a major challenge faced by the emergency departments globally affecting the quality of care provided to the patients. An increase in length of stay because of delay in the acquisition of diagnostic tests like MRI may affect throughput and cause overcrowding in the emergency rooms ultimately resulting in an increased cost.

The challenge to balance the risk of missing a serious etiology like stroke against putting the financial burden on patients and their families is even bigger in low-income and low-middle-income countries, where the primary source of payment is out of pocket payment. To the best of our knowledge, there are no studies from the emergency rooms of either a low-income or a low-middle-income country which estimated the frequency and cost involved in the neuro-imaging of patients with vertigo.

Hence, this study aimed to determine the frequency of neuro-imaging and the prevalence of positive findings in patients with vertigo visiting an emergency room of a low-middle-income-country (LMIC), Pakistan. In addition, we also analyzed the cost-utility of neuro-imaging in these patients.

## METHODS

This is a retrospective cross-sectional study conducted at the emergency department of the Aga Khan University Hospital (AKUH), a 550 bedded-tertiary care teaching facility located in Karachi, Pakistan, having advanced neurological & radiological facilities available. The emergency department of AKUH is a 62-bedded facility that receives 84,000 patients annually. Institutional Ethical Review Committee’s approval was obtained prior to the data collection.

### Inclusion & Exclusion criteria

Data of patients having age 18 years and above who presented to the emergency room with complaints of vertigo and dizziness were collected. Patients who went underwent neuro-imaging for reasons other than vertigo, like trauma or injury, were excluded from the study. Data was collected through convenient sampling method.

### Data Collection

The data were collected retrospectively by reviewing the records of patients who visited the emergency room with vertigo during the last 20 years (2000-2020). The selection process of participants was made via a system-based approach in which vertigo and dizziness coded triage slips were extracted as per the ICD 20 classification. Data were collected from patient care software (Patient Care Inquiry- PCI) by entering the medical record number of patients extracted with the coded complaint. Discharge summaries were reviewed, and further filtration of patients to be included was done. A pilot of 10 patients was done initially with the data collectors, and the questionnaire was modified according to the gaps identified.

Data was collected in a pre-designed questionnaire in which variables including patient’s demographics, duration of complaints, co-morbidities, indication & results of neuroimaging with the patient final disposition were included. The questionnaire was reviewed for missing information or, in the case of contradictory information provided. This was done to ensure the sampling quality, and the data entered.

### Data Analysis

The cost utility analysis was performed by comparing the costs involved in neuroimaging in patients with positive findings as compared to those with negative findings. The data were analyzed using the Statistical Package for the Social Sciences (SPSS) Version 21.

### Ethical Approval

The study received institution ERC approval (ERC 2020-3345-10284).

## RESULTS

The study included 224 patients who met the inclusion criteria and presented to the emergency room with vertigo. The frequency was almost equally distributed between males and females (49.55% vs. 50.45%). The mean age of the patients was 60.89 ± 16.2 years, ranging between (23 to 99) years. Commonly associated co-morbid conditions in patients with vertigo coming to the emergency room were Hypertension (53.47%), Diabetes Mellitus (40.59%), and history of ischemic heart disease (16.34%). The symptoms commonly associated with vertigo were vomiting (29.46%), drowsiness (19.64%), and headache (17.86%). 159 (70.98%) patients went under neuroimaging. MRI was performed in 126 (76.83%) patients, C.T. scan was performed in 26 (15.85%) patients, whereas 12 (7.32%) patients underwent both a C.T. scan and MRI. (Table-I) More than 95% of the patients who presented to the emergency room with vertigo were hospitalized.

**Table-I:** Baseline, Demographics, and Clinical Characteristics in patients with vertigo.

	Frequency [%]
***Gender Distribution***	
Male	110 [49.6%]
Female	113 [50.4%]
***Brain Imaging***	
Yes	159 [70.98%]
No	65 [29.02%]
***Brain Imaging***	
CT	26 [15.85%]
MRI	126 [76.83%]
CT/MRI	12 [7.32%]
Total	164 [100%]
***Hospital Disposition***	
Ward	177 [79.02%]
SCU	27 [12.05%]
Discharge	9 [4.02%]
LAMA	2 [0.89%]
CCU	5 [2.23%]
Neuro	1 [0.45%]
ICU	3 [1.34%]
***Associated Complains***	
Nausea	18 [8.04%]
Vomiting	66 [29.46%]
Gait Disturbance	8 [3.57%]
Dizziness/Drowsy	44 [19.64%]
Headache	40 [17.86%]
HTN/ High BP	14 [6.25%]
Vertigo	207 [92.41%]
Fever	8 [3.57%]
Fall/Head Trauma/RTA	18 [8.04%]
Generalized Weakness/Decrease Oral Intake	12 [5.36%]
Drowsiness/Black out	6 [2.68%]
Abdominal Pain	5 [2.23%]
OtheRs.	64 [28.57%]
***Multiple Comorbidities***	
DM	82 [40.59%]
HTN	108 [53.47%]
IHD/CAD	33 [16.34%]
None	26 [12.87%]
OtheRs.	107 [52.97%]
***Image Findings***	
Infarct	37 [41.57%]
Haemorrhage	14 [15.73%]
SOL	5 [5.62%]
Meningeal Enhancement lumber puncture/cerebral edema	2 [2.25%]
Age Appropriate Changes	12 [13.48%]
Mets	6 [6.74%]
Old Ischaemic	2 [2.25%]
Old Infarct	2 [2.25%]
Global involutional brain changes	3 [3.37%]
OtheRs.	9 [10.11%]
***Age Groups***	
<30 years	13 [5.8%]
31-60 years	89 [39.7%]
>60 years	122 [54.5%]

Vomiting and drowsiness were the most significantly associated complaints with vertigo in patients who underwent neuroimaging (P= 0.021 & P= 0.002) (Table-II). Out of 159 patients who underwent neuroimaging, 64 patients (40.25%) had significant findings. The most common finding was acute infarct in 37 (41.57%) followed by hemorrhage in 14 (15.73%), metastasis in 06 (6.74%), space-occupying lesion (SOL) in 05 (5.62%), and meningeal enhancement in 02 (2.25%) of the patients. More than 90% of the patients were offered in-hospital admission (Table-III).

**Table-II:** Association of presenting complaints with vertigo who underwent with and without neuroimaging.

Characteristics	Total	Brain Imaging		p-value

		Yes	No	
Associated Complain	224	159	65	-
Total Sample Size	224	159	65	
Nausea	18 [8.04%]	12 [7.55%]	6 [9.23%]	0.674
Vomiting	66 [29.46%]	54 [33.96%]	12 [18.46%]	0.021*
Gait Disturbance	8 [3.57%]	8 [5.03%]	0 [0%]	0.066
Dizziness/Drowsy	44 [19.64%]	23 [14.47%]	21 [32.31%]	0.002*
Headache	40 [17.86%]	31 [19.5%]	9 [13.85%]	0.316
HTN/ High BP	14 [6.25%]	10 [6.29%]	4 [6.15%]	0.970
Vertigo	207 [92.41%]	146 [91.82%]	61 [93.85%]	0.604
Fever	8 [3.57%]	3 [1.89%]	5 [7.69%]	0.034*
Fall/Head Trauma/RTA	18 [8.04%]	15 [9.43%]	3 [4.62%]	0.229
G.Wekaness/Decrease Oral Intake	12 [5.36%]	3 [1.89%]	9 [13.85%]	<0.001*
Drowsiness/Black out	6 [2.68%]	2 [1.26%]	4 [6.15%]	0.039*
Abdominal Pain	5 [2.23%]	3 [1.89%]	2 [3.08%]	0.584
Others.	64 [28.57%]	42 [26.42%]	22 [33.85%]	0.264
Total	224 [100%]	159 [100%]	65 [100%]	----

**Table-III:** Comparison of braining imaging vs. image findings.

Characteristics	Total	Brain Imaging		p-value

		Yes	No	
Image Findings	224	159	65	-
Total Sample Size	89	85	4	
Infarct	37 [41.57%]	37 [43.53%]	0 [0%]	0.084
Haemorrhage	14 [15.73%]	14 [16.47%]	0 [0%]	0.377
SOL	5 [5.62%]	5 [5.88%]	0 [0%]	0.618
Meningeal Enhancement lumber puncture/cerebral edema	2 [2.25%]	2 [2.35%]	0 [0%]	0.756
Age-Appropriate Changes	12 [13.48%]	11 [12.94%]	1 [25%]	0.490
Mets	6 [6.74%]	5 [5.88%]	1 [25%]	0.136
Old Ischaemic	2 [2.25%]	1 [1.18%]	1 [25%]	0.002*
Old Infarct	2 [2.25%]	2 [2.35%]	0 [0%]	0.756
Global involutional brain changes	3 [3.37%]	2 [2.35%]	1 [25%]	0.014*
Others.	9 [10.11%]	9 [10.59%]	0 [0%]	0.492
Total	89 [100%]	85 [100%]	4 [100%]	----

The department’s total annual healthcare service cost was Rs.6,571,800 ($35912). A total of 61(38.4%) were positive, and 98(61.6%) were negative findings. For patients with negative brain findings, the expenditure was Rs.40,75,200 and Rs.24,96,600 were spent on patients with positive findings. The total favorable (positive) cost, which accounted for 61 patients, Rs.180,400 ($986) was spent on CT, while Rs.1,835,400 ($10030) was spent on MRI, & Rs.480,800 ($2627) spent on CT/MRI. Accordingly, it was seen that the vertigo patients take up Rs.2,496,600, with a total of 61 patients accounting for [93.85%] of the total annual cost of our department. Out of a total of 61 positive findings, 11 C.T. scan patients incurred the cost of Rs.180,400 ($986); among these 11 patients, five were diagnosed with Hemorrhage with a cost of Rs.82,000($448), followed by three who had Infarct with Rs.49,200 ($269) and remaining three patients who had SOL/Metastasis and Meningeal Enhancement with the same cost. Forty two patients did MRI; among these, 28 were diagnosed with an Infarct with a cost of Rs.1,223,600 ($6686), eight were Hemorrhage with the cost of Rs.349,600 ($1910), two were SOL with Rs.87,400 ($478)), four were Metastasis with Rs.174,800 ($955). Eight CT/MRI patients incurred the cost of Rs.480,800 ($2628), out of which six patients were diagnosed with Infarct (Table-IV).

**Table-IV:** Cost analysis of positive patients’ diagnosis with CT, MRI, and CT/MRI imaging

	CT		MRI		CT/MRI		Total	

Positive Patients	Cost=16400 (PKR)		Cost=43700 (PKR)		Cost=60100 (PKR)		Cost	

	Total amount	Absolute# Patients	Total amount	Absolute# Patients	Total amount	Absolute# Patients	Total amount	Cumulative
Infarct	Rs.49,200	3	Rs.1,223,600	28	Rs.360,600	6	Rs.1,633,400	37
Haemorrhage	Rs.82,000	5	Rs.349,600	8	Rs.60,100	1	Rs.491,700	14
SOL	Rs.16,400	1	Rs.87,400	2	Rs.120,200	2	Rs.224,000	5
Meningeal Enhancement lumber puncture/ cerebral edema	Rs.16,400	1	Rs.43,700	1	------	------	Rs.60,100	2
Mets	Rs.16,400	1	Rs.174,800	4	Rs.60,100	1	Rs.251,300	6

Total	Rs.180,400	11	Rs.1,835,400	42	Rs.480,800	8	Rs.2,496,600	61

**Fig.1:**
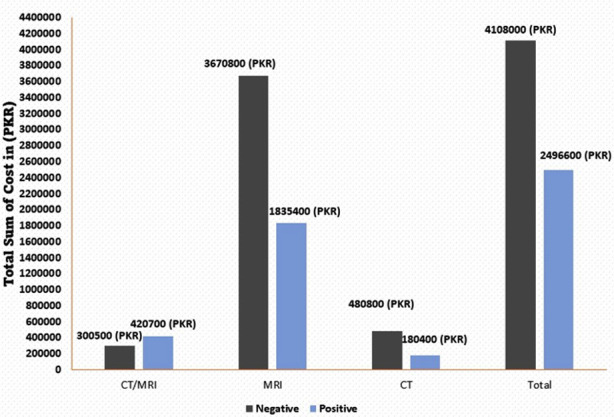
Comparison of a cost analysis of vertigo patients’ diagnosis with positive veRs.us negative neuroimaging findings among CT/MRI, MRI, and CT scan.

According to (Fig.1), it was observed that out of a total of 159 patients, 98 negative patients significantly found a higher cost of Rs.4,108,000 ($ 22449) as compared to positive patients at Rs.2,496,600 ($13642). Furthermore, out of 126 patients who underwent an MRI, 84(85.7%) had clinically insignificant findings that costed them Rs.3,670,800, as compared to clinically significant findings in 42 patients with a cost of Rs.1,835,400 ($20060).

## DISCUSSION

Vertigo and dizziness are not only one of the common presenting complaints to the emergency rooms but also affect work productivity and have an impact on the economic burden on healthcare systems.15 One study showed an overall impact on work productivity in patients with peripheral vertigo to be 15.35+/- 6.11 days.16 Another study showed 63.3% of the affected patients losing their workdays, 4.6% changing their jobs and 5.7% giving up their employment because of daily symptoms of dizziness.17 This impact on work productivity and economic burden on healthcare systems can be attributed to the severity of an underlying etiology like a neurological or cardiac disorder. Our study showed that neurological diseases including acute infarcts (37), hemorrhage (14), metastasis (06), space occupying lesions (05) and meningeal enhancement (02) contributed 40% of the burden of the patients with vertigo who underwent neuroimaging. Data from the low-income and low-middle income countries is scarce, however a modest burden of neurological and cardiac diseases contributing to vertigo in emergency room visits has been highlighted in various studies.18-20 The fear of missing these significant and potentially fatal neurological etiologies like infarcts, hemorrhage and malignancies may cause a cognitive bias that encourage emergency physicians to over-investigate in terms of obtaining neuroimaging in patients with vertigo. In our study, a significant number of patients (70.98 %) visiting to the emergency room with vertigo underwent neuroimaging. In addition, more than 90% of our patients have been advised admission for further evaluation. This resulted in the addition of cost and resulted in an economic burden on patients belonging to a low-middle-income country.

In our study, we observed that out of 224 cases undergoing neuroimaging due to vertigo, patients with negative findings faced significantly higher costs, totalling Rs. 4,108,000 ($22,449), while those with positive results incurred Rs. 2,496,600 ($13,642). This financial burden aligns with similar studies investigating the economic implications of diagnosing and managing vertigo. For instance, Becares-Martinez C et al. reported that out of 493 cases, 286 imaging tests for vertigo cost 56,741 euros ($60,400), with a positive test amounting to 1,576 euros ($1,670).21 Additionally, our study’s proportion of patients undergoing neuroimaging (70.98%) surpasses figures from related research conducted in a Turkish emergency department (28%)22. A systematic review which included studies from developed countries identified three main drivers of increased direct costs including excessive use of diagnostic imaging and excessive use of emergency care.15 In addition, the duration of hospitalization is also associated with an increase in cost, that is increase in the length of stay results in higher costs.23 This financial burden can have a direct impact on the patients and their families especially in low and low-middle-income countries where out-of-pocket payment is the primary of medical-bill payments instead of third-party or medical insurance.

These collective findings underscore the substantial financial strain faced by patients and families, particularly in regions where out-of-pocket payments prevail as the primary mode of medical expense coverage. Considering Pakistan’s approximate household income per capita of $508 in 2019, it’s evident that the expenses associated with obtaining neuroimaging pose a significant financial challenge for affected individuals and their families.24

This highlights the need of a cost-effective and sustainable intervention for emergency physicians in context of resource limited countries like Pakistan that can help them to make well-informed decisions while requesting costly investigations like MRI in patients with vertigo. This may include developing a clinical decision rule (CDR) specifically for emergency physicians that may help them to efficiently utilize resources like a CT scan or an MRI in patients with vertigo. This need is also endorsed by a multi-national survey which showed that emergency physicians consider vertigo as one of the high-priority area for developing future CDRs that may help in the diagnosis and management of the disease.25 Additionally, these interventions may not only help to reduce the economic burden but also assist in diagnosing serious conditions like stroke, which may have a likelihood of being overlooked during a patient’s initial medical evaluation.26-28

### Limitations

Although there were significant findings in terms of the frequency of neuroimaging, the frequency of positive neurological findings and the costs involved for neuroimaging in perspective of a low-middle-income country like Pakistan in our study, there were some limitations as well. First, this was a retrospective single center study and lacks external validity. A prospective multicenter study is required to give a holistic picture so that results can be generalized to a larger population. Second, we did not look into the final diagnosis at the hospital discharge which could have helped us to determine the prevalence of neurological diseases in patients with vertigo and their association with the neuro-imaging findings. Moreover, comparing the costs in terms of national economic indicators like the GDP could have helped to give a better insight in terms of the financial burden beared by the patients in a low-middle-income country.

## CONCLUSION

The frequency of obtaining neuro-imaging tests in patients with vertigo were significantly high in the emergency room of a tertiary care hospital in Karachi Pakistan. Although, the frequency of patients diagnosed with a neurological disease was more as compared to some of the other studies, there was a significant financial burden associated with neuroimaging especially for a low-middle-income country like Pakistan. This study may prove to be a stepping-stone for researchers. to design more robust studies to assess the need of neuro-imaging tests in patients with vertigo coming to the emergency room.

### Authors Contributions:

**NB** developed the research protocol and designed it.

**AR** did the statistical analysis and drafted the results section, official responsibility for the overall integrity of the manuscript and attest that all statements in the manuscript are true to his knowledge.

**NK** drafted the protocol and prepared the data collection tool and did the final review of the draft and proofread.

**MI** did the final review of the draft and proofread it.

All authors reviewed the manuscript.
